# (3*R*,6*R*,12*R*,20*S*,24*S*)-3,6,12-Triacetyl-20,24-ep­oxy­dammarane-3,6,12,25-tetra­ol

**DOI:** 10.1107/S1600536810050749

**Published:** 2010-12-11

**Authors:** Huan-Mei Guo, Liang Wang, Nan Wang, Jiang-Feng Zhang, Qing-Guo Meng

**Affiliations:** a Microscale Science Institute, Weifang University, Weifang 261061, People’s Republic of China; b School of Pharmacy, Yantai University, Yantai 264005, People’s Republic of China

## Abstract

The title compound, C_36_H_58_O_8_, was prepared from 20(*S*)-protopanaxatriol, which was degraded from *Panax quinquefolium saponin* with sodium in glycerine, extracted and seperated by flash chromatography. Three six-membered rings are in chair conformations, the five-membered ring is in an envelope form and the tetra­hydro­furan ring has a conformation inter­mediate between half-chair and envelope. In the crystal, mol­ecules are linked by O—H⋯O hydrogen bonds, and C—H⋯O contacts also occur. The absolute structure was assigned on the basis of the synthesis.

## Related literature

For background to the medicinal uses of Araliaceae-type natural products, see: Shibata *et al.* (1985 ▶); Takano *et al.* (1999 ▶); Yu *et al.* (2007 ▶); Wang *et al.* (2010 ▶). For related structures, see: Iljin *et al.* (1982 ▶); Shi *et al.* (1992 ▶).
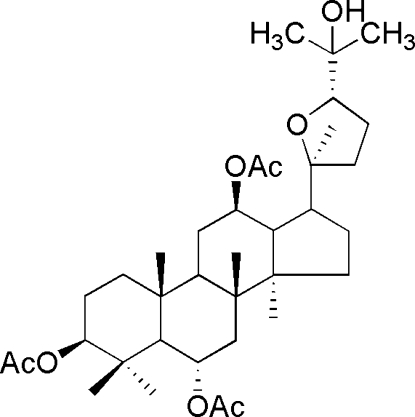


## Experimental

### 

#### Crystal data

C_36_H_58_O_8_
*M* *_r_* = 618.82
Orthorhombic, 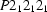

*a* = 7.6936 (11) Å
*b* = 16.151 (2) Å
*c* = 28.439 (4) Å
*V* = 3533.7 (9) Å^3^
*Z* = 4
Mo *K*α radiation
μ = 0.08 mm^−1^
*T* = 298 K
0.41 × 0.20 × 0.10 mm

#### Data collection

Bruker SMART CCD diffractometer
18696 measured reflections
3738 independent reflections
2746 reflections with *I* > 2σ(*I*)
*R*_int_ = 0.044

#### Refinement

*R*[*F* ^2^ > 2σ(*F* ^2^)] = 0.045
*wR*(*F* ^2^) = 0.115
*S* = 1.04
3738 reflections
409 parameters
H-atom parameters constrained
Δρ_max_ = 0.20 e Å^−3^
Δρ_min_ = −0.15 e Å^−3^

### 

Data collection: *SMART* (Bruker, 1997 ▶); cell refinement: *SAINT* (Bruker, 1997 ▶); data reduction: *SAINT*; program(s) used to solve structure: *SHELXS97* (Sheldrick, 2008 ▶); program(s) used to refine structure: *SHELXL97* (Sheldrick, 2008 ▶); molecular graphics: *SHELXTL* (Sheldrick, 2008 ▶); software used to prepare material for publication: *SHELXTL*.

## Supplementary Material

Crystal structure: contains datablocks global, I. DOI: 10.1107/S1600536810050749/hb5749sup1.cif

Structure factors: contains datablocks I. DOI: 10.1107/S1600536810050749/hb5749Isup2.hkl

Additional supplementary materials:  crystallographic information; 3D view; checkCIF report

## Figures and Tables

**Table 1:** Hydrogen-bond geometry (Å, °)

*D*—H⋯*A*	*D*—H	H⋯*A*	*D*⋯*A*	*D*—H⋯*A*
O1—H1⋯O8^i^	0.82	2.36	3.050 (4)	142
C8—H8⋯O8^i^	0.98	2.58	3.526 (4)	161
C35—H35*C*⋯O6^ii^	0.96	2.45	3.403 (5)	172

Symmetry codes: (i) 
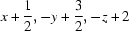
; (ii) 
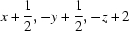
.

**Fig. 1.**
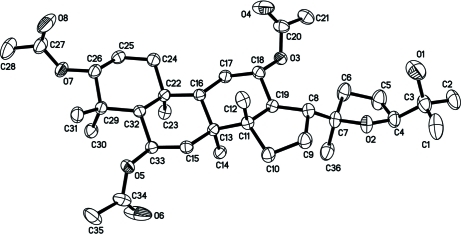
The molecular structure of the title compound with displacement ellipsoids drawn at the 30% probability level.

## supplementary crystallographic information

### Comment

Both Panax ginseng and Panax quinquefolium, belonging to the Araliaceae, are well known traditional medicinal herbs. They are used as tonics and the treatment for diseases, such as tumor and myocardial ischemia. Panax ginseng contains numbers of saponins, called ginsenoside, including an oleanolic acid-type saponin in addition to the major protopanaxadiol and protopanaxatriol-type saponins (Shibata *et al.*,1985). Panax quinquefolium contains an ocotillol-type (20*S*, 24*R*-epoxyside) saponin with high anti-tumor activity (Takano *et al.*,1999), as well as oleanolic acid-type saponin, protopanaxadiol and protopanaxatriol-type saponins. (3*R*,6*R*,12*R*,20S,24*S*)-20,24-epoxy-dammarane-3,6,12,25-tetraol and (3*R*,6*R*,12*R*,20S,24*R*)-20,24-epoxy-dammarane-3,12,25-triol are found to possess cardioprotective effect on myocardial injury induced by isoproterenol in rats (Yu *et al.*,2007; Wang *et al.*,2010). As part of our ongoing investigation of ocotillol-type compound and their cardioprotective effect on myocardial injury, we report here the crystal structure of the title compound, (I).

In the molecule, all bond lengths and angles are within normal ranges (Shi *et al.*, 1992; Iljin *et al.*, 1982) Rings A(C11,C13,C16,C17,C18,C19), B(C13,C15,C16,C22,C32,C33), and C(C22,C24,C25,C26,C29,C32)are in chair conformations. Ring D(8,C9,C10,C11,C19) has an envelope form with C11 as the out-of-plane atom. The tetrahydrofuran ring has a conformation intermediate between half-chair and envelope forms. In the crystal, O—H···O hydrogen bonds occur.

### Experimental

20(S)-protopanaxatriol was degraded from Panax quinquefolium saponin with sodium in glycerine at about 200°C and seperated by flash chromatography. (3*R*,6*R*,12*R*,20S,24*S*)-3,6,12-acetyl-20,24-epoxy-dammarane- 3,6,12,25-tetraol was synthesized from 20(S)-protopanaxatriol in the presence of *N*,*N*-dimethylaminopyridine, pyridine and acetic anhydride. The esters were oxidized by *m*-CPBA and seperated by flash chromatography. Finally, the crystals were dried at room temperature. Colourless blocks of (I) were obtained by recrystallization from ethyl acetate and petroleum ether (60–90°C) at room temperature.

### Refinement

Anomalous dispersion was negligible and Friedel pairs were merged before refinement. All H atoms were fixed geometrically and allowed to ride on their attached atoms, with C—H distances in the range 0.93–0.97 Å, and with *U*_iso_(H) = 1.2*U*_eq_ of the parent atoms.

### Crystal data

**Table:**

C_36_H_58_O_8_	*F*(000) = 1352
*M**_r_* = 618.82	*D*_x_ = 1.163 Mg m^−^^3^
Orthorhombic, *P*2_1_2_1_2_1_	Mo *K*α radiation, λ = 0.71073 Å
Hall symbol: P 2ac 2ab	Cell parameters from 3209 reflections
*a* = 7.6936 (11) Å	θ = 2.5–20.5°
*b* = 16.151 (2) Å	µ = 0.08 mm^−^^1^
*c* = 28.439 (4) Å	*T* = 298 K
*V* = 3533.7 (9) Å^3^	Block, colourless
*Z* = 4	0.41 × 0.20 × 0.10 mm

### Data collection

**Table:**

Bruker SMART CCD diffractometer	2746 reflections with *I* > 2σ(*I*)
Radiation source: fine-focus sealed tube	*R*_int_ = 0.044
graphite	θ_max_ = 25.5°, θ_min_ = 1.9°
φ and ω scans	*h* = −9→9
18696 measured reflections	*k* = −19→16
3738 independent reflections	*l* = −25→34

### Refinement

**Table:**

Refinement on *F*^2^	Primary atom site location: structure-invariant direct methods
Least-squares matrix: full	Secondary atom site location: difference Fourier map
*R*[*F*^2^ > 2σ(*F*^2^)] = 0.045	Hydrogen site location: inferred from neighbouring sites
*wR*(*F*^2^) = 0.115	H-atom parameters constrained
*S* = 1.04	*w* = 1/[σ^2^(*F*_o_^2^) + (0.0618*P*)^2^ + 0.1172*P*] where *P* = (*F*_o_^2^ + 2*F*_c_^2^)/3
3738 reflections	(Δ/σ)_max_ = 0.001
409 parameters	Δρ_max_ = 0.20 e Å^−^^3^
0 restraints	Δρ_min_ = −0.15 e Å^−^^3^

### Special details

**Table:**

Geometry. All e.s.d.'s (except the e.s.d. in the dihedral angle between two l.s. planes) are estimated using the full covariance matrix. The cell e.s.d.'s are taken into account individually in the estimation of e.s.d.'s in distances, angles and torsion angles; correlations between e.s.d.'s in cell parameters are only used when they are defined by crystal symmetry. An approximate (isotropic) treatment of cell e.s.d.'s is used for estimating e.s.d.'s involving l.s. planes.
Refinement. Refinement of *F*^2^ against ALL reflections. The weighted *R*-factor *wR* and goodness of fit *S* are based on *F*^2^, conventional *R*-factors *R* are based on *F*, with *F* set to zero for negative *F*^2^. The threshold expression of *F*^2^ > σ(*F*^2^) is used only for calculating *R*-factors(gt) *etc*. and is not relevant to the choice of reflections for refinement. *R*-factors based on *F*^2^ are statistically about twice as large as those based on *F*, and *R*- factors based on ALL data will be even larger.

### Fractional atomic coordinates and isotropic or equivalent isotropic displacement parameters (Å^2^)

**Table:**

	*x*	*y*	*z*	*U*_iso_*/*U*_eq_	
C1	1.1476 (7)	0.7885 (3)	0.68525 (15)	0.1027 (16)	
H1A	1.2080	0.8385	0.6771	0.154*	
H1B	1.1758	0.7460	0.6629	0.154*	
H1C	1.1820	0.7711	0.7161	0.154*	
C2	0.8932 (7)	0.8297 (2)	0.63522 (11)	0.0903 (14)	
H2A	0.7714	0.8424	0.6358	0.136*	
H2B	0.9138	0.7849	0.6137	0.136*	
H2C	0.9573	0.8776	0.6253	0.136*	
C3	0.9526 (7)	0.8043 (2)	0.68452 (12)	0.0759 (12)	
C4	0.8559 (5)	0.7273 (2)	0.69971 (11)	0.0632 (9)	
H4	0.8821	0.6828	0.6774	0.076*	
C5	0.6602 (6)	0.7335 (3)	0.70503 (13)	0.0886 (13)	
H5A	0.6023	0.6920	0.6860	0.106*	
H5B	0.6193	0.7878	0.6956	0.106*	
C6	0.6258 (5)	0.7188 (3)	0.75713 (12)	0.0728 (11)	
H6A	0.6216	0.7706	0.7743	0.087*	
H6B	0.5175	0.6892	0.7619	0.087*	
C7	0.7806 (5)	0.6667 (2)	0.77218 (11)	0.0579 (9)	
C8	0.8360 (4)	0.6771 (2)	0.82373 (10)	0.0491 (8)	
H8	0.8490	0.7364	0.8304	0.059*	
C9	1.0135 (4)	0.6336 (2)	0.83290 (12)	0.0604 (9)	
H9A	1.0605	0.6114	0.8039	0.072*	
H9B	1.0966	0.6723	0.8461	0.072*	
C10	0.9758 (4)	0.5640 (2)	0.86767 (11)	0.0535 (8)	
H10A	1.0781	0.5507	0.8861	0.064*	
H10B	0.9368	0.5144	0.8515	0.064*	
C11	0.8317 (4)	0.59958 (17)	0.89865 (10)	0.0427 (7)	
C12	0.9179 (4)	0.66713 (19)	0.92930 (11)	0.0560 (8)	
H12A	1.0206	0.6451	0.9438	0.084*	
H12B	0.8379	0.6847	0.9532	0.084*	
H12C	0.9485	0.7136	0.9099	0.084*	
C13	0.7242 (4)	0.53600 (17)	0.92850 (9)	0.0397 (7)	
C14	0.6515 (4)	0.46700 (18)	0.89651 (10)	0.0498 (7)	
H14A	0.7338	0.4550	0.8721	0.075*	
H14B	0.5441	0.4850	0.8827	0.075*	
H14C	0.6315	0.4180	0.9148	0.075*	
C15	0.8380 (4)	0.49472 (18)	0.96601 (9)	0.0439 (7)	
H15A	0.9187	0.4574	0.9507	0.053*	
H15B	0.9055	0.5370	0.9819	0.053*	
C16	0.5777 (4)	0.58613 (17)	0.95394 (9)	0.0397 (6)	
H16	0.6401	0.6290	0.9716	0.048*	
C17	0.4668 (4)	0.63455 (19)	0.91841 (10)	0.0483 (7)	
H17A	0.4007	0.5956	0.8995	0.058*	
H17B	0.3847	0.6689	0.9354	0.058*	
C18	0.5747 (4)	0.68930 (17)	0.88586 (10)	0.0457 (7)	
H18	0.6304	0.7333	0.9042	0.055*	
C19	0.7118 (4)	0.63893 (18)	0.86098 (9)	0.0409 (7)	
H19	0.6511	0.5931	0.8455	0.049*	
C20	0.3777 (4)	0.7956 (2)	0.86171 (13)	0.0606 (9)	
C21	0.2781 (5)	0.8289 (2)	0.82093 (13)	0.0793 (12)	
H21A	0.1806	0.8603	0.8322	0.119*	
H21B	0.2374	0.7839	0.8019	0.119*	
H21C	0.3521	0.8641	0.8025	0.119*	
C22	0.4723 (4)	0.53757 (17)	0.99232 (9)	0.0427 (7)	
C23	0.3525 (4)	0.4714 (2)	0.97065 (10)	0.0537 (8)	
H23A	0.2590	0.4596	0.9920	0.081*	
H23B	0.4178	0.4218	0.9650	0.081*	
H23C	0.3059	0.4916	0.9415	0.081*	
C24	0.3551 (4)	0.5997 (2)	1.01821 (11)	0.0567 (8)	
H24A	0.4240	0.6474	1.0273	0.068*	
H24B	0.2652	0.6187	0.9969	0.068*	
C25	0.2702 (4)	0.5633 (2)	1.06176 (11)	0.0612 (9)	
H25A	0.1995	0.6051	1.0770	0.073*	
H25B	0.1951	0.5177	1.0528	0.073*	
C26	0.4069 (4)	0.53286 (19)	1.09541 (10)	0.0526 (8)	
H26	0.4727	0.5807	1.1069	0.063*	
C27	0.3014 (5)	0.5363 (3)	1.17474 (12)	0.0698 (10)	
C28	0.2086 (7)	0.4860 (3)	1.21129 (12)	0.1058 (16)	
H28A	0.2609	0.4322	1.2133	0.159*	
H28B	0.0885	0.4804	1.2027	0.159*	
H28C	0.2171	0.5133	1.2412	0.159*	
C29	0.5362 (4)	0.46892 (18)	1.07605 (9)	0.0454 (7)	
C30	0.4519 (5)	0.38367 (19)	1.07045 (11)	0.0591 (9)	
H30A	0.4195	0.3627	1.1008	0.089*	
H30B	0.5330	0.3464	1.0560	0.089*	
H30C	0.3502	0.3884	1.0511	0.089*	
C31	0.6807 (5)	0.4635 (2)	1.11355 (10)	0.0581 (9)	
H31A	0.6294	0.4641	1.1443	0.087*	
H31B	0.7577	0.5099	1.1103	0.087*	
H31C	0.7449	0.4130	1.1094	0.087*	
C32	0.6090 (4)	0.50400 (16)	1.02858 (9)	0.0398 (6)	
H32	0.6786	0.5523	1.0375	0.048*	
C33	0.7351 (4)	0.44676 (16)	1.00231 (9)	0.0407 (7)	
H33	0.6682	0.4034	0.9863	0.049*	
C34	0.9002 (5)	0.3292 (2)	1.02639 (14)	0.0645 (9)	
C35	1.0098 (5)	0.2951 (2)	1.06456 (14)	0.0847 (13)	
H35A	0.9449	0.2543	1.0818	0.127*	
H35B	1.0441	0.3389	1.0854	0.127*	
H35C	1.1114	0.2698	1.0513	0.127*	
C36	0.7616 (6)	0.5761 (2)	0.75780 (12)	0.0797 (12)	
H36A	0.7418	0.5727	0.7245	0.120*	
H36B	0.6650	0.5519	0.7742	0.120*	
H36C	0.8660	0.5466	0.7656	0.120*	
O1	0.9096 (7)	0.87108 (16)	0.71447 (9)	0.1250 (15)	
H1	0.9242	0.8572	0.7419	0.188*	
O2	0.9193 (3)	0.70347 (14)	0.74538 (7)	0.0628 (6)	
O3	0.4598 (3)	0.72584 (12)	0.85100 (7)	0.0506 (5)	
O4	0.3818 (5)	0.82663 (17)	0.90006 (10)	0.1007 (10)	
O5	0.8577 (3)	0.40815 (12)	1.03405 (6)	0.0487 (5)	
O6	0.8527 (5)	0.29176 (17)	0.99273 (14)	0.1268 (14)	
O7	0.3183 (3)	0.49444 (14)	1.13542 (7)	0.0617 (6)	
O8	0.3532 (6)	0.60487 (19)	1.18027 (10)	0.1177 (13)	

### Atomic displacement parameters (Å^2^)

**Table:**

	*U*^11^	*U*^22^	*U*^33^	*U*^12^	*U*^13^	*U*^23^
C1	0.115 (4)	0.121 (4)	0.072 (3)	−0.054 (4)	−0.001 (3)	0.025 (3)
C2	0.153 (4)	0.071 (2)	0.0471 (19)	−0.024 (3)	−0.007 (3)	0.0122 (18)
C3	0.124 (4)	0.061 (2)	0.043 (2)	−0.021 (2)	−0.005 (2)	0.0010 (16)
C4	0.084 (3)	0.064 (2)	0.0417 (18)	−0.008 (2)	0.0018 (18)	0.0070 (16)
C5	0.079 (3)	0.128 (4)	0.059 (2)	0.002 (3)	−0.005 (2)	0.017 (2)
C6	0.064 (2)	0.108 (3)	0.0461 (19)	−0.002 (2)	−0.0051 (18)	0.0076 (19)
C7	0.058 (2)	0.069 (2)	0.0470 (18)	−0.0120 (18)	0.0071 (16)	0.0079 (16)
C8	0.0502 (18)	0.0514 (18)	0.0458 (17)	−0.0065 (16)	−0.0007 (15)	0.0055 (14)
C9	0.0511 (19)	0.070 (2)	0.060 (2)	−0.0027 (18)	0.0056 (16)	0.0103 (17)
C10	0.0380 (15)	0.066 (2)	0.0564 (18)	0.0029 (15)	0.0056 (15)	0.0109 (16)
C11	0.0368 (15)	0.0464 (16)	0.0449 (16)	−0.0026 (14)	−0.0012 (13)	0.0029 (13)
C12	0.0494 (18)	0.0583 (19)	0.060 (2)	−0.0145 (16)	−0.0097 (17)	0.0103 (15)
C13	0.0372 (14)	0.0447 (16)	0.0373 (15)	0.0017 (13)	0.0012 (12)	−0.0013 (12)
C14	0.0547 (17)	0.0498 (17)	0.0449 (16)	−0.0022 (16)	0.0055 (15)	−0.0046 (14)
C15	0.0401 (15)	0.0451 (16)	0.0465 (17)	0.0040 (13)	0.0002 (14)	0.0014 (13)
C16	0.0401 (15)	0.0397 (15)	0.0394 (15)	0.0027 (13)	−0.0035 (13)	−0.0013 (12)
C17	0.0445 (16)	0.0540 (18)	0.0465 (17)	0.0083 (15)	0.0012 (14)	0.0032 (14)
C18	0.0453 (16)	0.0471 (17)	0.0448 (16)	0.0043 (14)	−0.0082 (14)	0.0046 (13)
C19	0.0396 (15)	0.0438 (16)	0.0393 (15)	−0.0017 (13)	−0.0029 (13)	−0.0011 (13)
C20	0.060 (2)	0.053 (2)	0.069 (2)	0.0124 (18)	−0.0128 (18)	−0.0024 (17)
C21	0.081 (3)	0.069 (2)	0.087 (3)	0.024 (2)	−0.011 (2)	0.016 (2)
C22	0.0408 (15)	0.0469 (16)	0.0403 (15)	0.0018 (14)	0.0019 (13)	−0.0021 (13)
C23	0.0424 (16)	0.070 (2)	0.0489 (18)	−0.0064 (17)	−0.0022 (15)	0.0016 (16)
C24	0.0553 (19)	0.064 (2)	0.0510 (18)	0.0154 (18)	0.0026 (16)	0.0064 (15)
C25	0.059 (2)	0.068 (2)	0.057 (2)	0.0167 (18)	0.0169 (17)	0.0012 (17)
C26	0.0605 (19)	0.0519 (18)	0.0455 (17)	−0.0080 (17)	0.0132 (16)	−0.0030 (14)
C27	0.080 (3)	0.078 (3)	0.052 (2)	−0.010 (2)	0.0204 (19)	−0.0053 (19)
C28	0.129 (4)	0.124 (4)	0.065 (2)	−0.038 (3)	0.040 (3)	−0.008 (2)
C29	0.0515 (17)	0.0460 (17)	0.0388 (15)	−0.0018 (15)	0.0041 (14)	0.0001 (13)
C30	0.071 (2)	0.0522 (19)	0.0540 (19)	−0.0110 (18)	0.0091 (17)	0.0003 (15)
C31	0.070 (2)	0.061 (2)	0.0425 (17)	−0.0027 (19)	−0.0006 (16)	0.0040 (15)
C32	0.0438 (16)	0.0365 (14)	0.0391 (15)	−0.0028 (13)	−0.0030 (13)	−0.0028 (12)
C33	0.0394 (15)	0.0418 (16)	0.0409 (16)	0.0024 (13)	−0.0037 (13)	−0.0024 (13)
C34	0.053 (2)	0.049 (2)	0.092 (3)	0.0138 (17)	−0.001 (2)	0.0021 (19)
C35	0.067 (2)	0.071 (2)	0.116 (3)	0.022 (2)	0.011 (2)	0.042 (2)
C36	0.102 (3)	0.082 (3)	0.055 (2)	−0.027 (3)	0.016 (2)	−0.0087 (19)
O1	0.247 (5)	0.0678 (17)	0.0601 (16)	−0.010 (3)	−0.016 (3)	−0.0145 (13)
O2	0.0642 (14)	0.0761 (15)	0.0481 (12)	−0.0093 (13)	0.0046 (12)	0.0153 (11)
O3	0.0531 (12)	0.0510 (12)	0.0478 (12)	0.0099 (11)	−0.0038 (10)	0.0040 (9)
O4	0.125 (2)	0.0804 (18)	0.097 (2)	0.0432 (18)	−0.036 (2)	−0.0276 (17)
O5	0.0498 (12)	0.0464 (12)	0.0500 (12)	0.0058 (10)	−0.0068 (10)	0.0058 (9)
O6	0.136 (3)	0.076 (2)	0.168 (3)	0.047 (2)	−0.066 (3)	−0.054 (2)
O7	0.0776 (15)	0.0617 (14)	0.0458 (12)	−0.0106 (12)	0.0191 (12)	−0.0055 (10)
O8	0.186 (3)	0.089 (2)	0.0789 (19)	−0.046 (3)	0.064 (2)	−0.0343 (16)

### Geometric parameters (Å, °)

**Table:**

C1—C3	1.522 (7)	C18—C19	1.509 (4)
C1—H1A	0.9600	C18—H18	0.9800
C1—H1B	0.9600	C19—H19	0.9800
C1—H1C	0.9600	C20—O4	1.201 (4)
C2—C3	1.531 (5)	C20—O3	1.327 (4)
C2—H2A	0.9600	C20—C21	1.491 (5)
C2—H2B	0.9600	C21—H21A	0.9600
C2—H2C	0.9600	C21—H21B	0.9600
C3—O1	1.414 (5)	C21—H21C	0.9600
C3—C4	1.511 (5)	C22—C24	1.537 (4)
C4—O2	1.440 (4)	C22—C23	1.540 (4)
C4—C5	1.516 (6)	C22—C32	1.570 (4)
C4—H4	0.9800	C23—H23A	0.9600
C5—C6	1.524 (5)	C23—H23B	0.9600
C5—H5A	0.9700	C23—H23C	0.9600
C5—H5B	0.9700	C24—C25	1.519 (4)
C6—C7	1.520 (5)	C24—H24A	0.9700
C6—H6A	0.9700	C24—H24B	0.9700
C6—H6B	0.9700	C25—C26	1.504 (4)
C7—O2	1.439 (4)	C25—H25A	0.9700
C7—C36	1.527 (5)	C25—H25B	0.9700
C7—C8	1.536 (4)	C26—O7	1.464 (3)
C8—C19	1.554 (4)	C26—C29	1.536 (4)
C8—C9	1.558 (5)	C26—H26	0.9800
C8—H8	0.9800	C27—O8	1.187 (4)
C9—C10	1.525 (4)	C27—O7	1.313 (4)
C9—H9A	0.9700	C27—C28	1.500 (5)
C9—H9B	0.9700	C28—H28A	0.9600
C10—C11	1.529 (4)	C28—H28B	0.9600
C10—H10A	0.9700	C28—H28C	0.9600
C10—H10B	0.9700	C29—C30	1.531 (4)
C11—C12	1.546 (4)	C29—C31	1.543 (4)
C11—C19	1.550 (4)	C29—C32	1.567 (4)
C11—C13	1.568 (4)	C30—H30A	0.9600
C12—H12A	0.9600	C30—H30B	0.9600
C12—H12B	0.9600	C30—H30C	0.9600
C12—H12C	0.9600	C31—H31A	0.9600
C13—C15	1.532 (4)	C31—H31B	0.9600
C13—C14	1.543 (4)	C31—H31C	0.9600
C13—C16	1.565 (4)	C32—C33	1.534 (4)
C14—H14A	0.9600	C32—H32	0.9800
C14—H14B	0.9600	C33—O5	1.447 (3)
C14—H14C	0.9600	C33—H33	0.9800
C15—C33	1.514 (4)	C34—O6	1.190 (4)
C15—H15A	0.9700	C34—O5	1.334 (4)
C15—H15B	0.9700	C34—C35	1.481 (5)
C16—C17	1.537 (4)	C35—H35A	0.9600
C16—C22	1.570 (4)	C35—H35B	0.9600
C16—H16	0.9800	C35—H35C	0.9600
C17—C18	1.526 (4)	C36—H36A	0.9600
C17—H17A	0.9700	C36—H36B	0.9600
C17—H17B	0.9700	C36—H36C	0.9600
C18—O3	1.453 (3)	O1—H1	0.8200
			
C3—C1—H1A	109.5	C19—C18—H18	109.6
C3—C1—H1B	109.5	C17—C18—H18	109.6
H1A—C1—H1B	109.5	C18—C19—C11	108.2 (2)
C3—C1—H1C	109.5	C18—C19—C8	122.4 (3)
H1A—C1—H1C	109.5	C11—C19—C8	105.5 (2)
H1B—C1—H1C	109.5	C18—C19—H19	106.6
C3—C2—H2A	109.5	C11—C19—H19	106.6
C3—C2—H2B	109.5	C8—C19—H19	106.6
H2A—C2—H2B	109.5	O4—C20—O3	123.4 (3)
C3—C2—H2C	109.5	O4—C20—C21	124.7 (3)
H2A—C2—H2C	109.5	O3—C20—C21	111.9 (3)
H2B—C2—H2C	109.5	C20—C21—H21A	109.5
O1—C3—C4	109.9 (3)	C20—C21—H21B	109.5
O1—C3—C1	110.5 (4)	H21A—C21—H21B	109.5
C4—C3—C1	110.1 (4)	C20—C21—H21C	109.5
O1—C3—C2	106.1 (3)	H21A—C21—H21C	109.5
C4—C3—C2	109.6 (3)	H21B—C21—H21C	109.5
C1—C3—C2	110.6 (4)	C24—C22—C23	107.1 (2)
O2—C4—C3	108.1 (3)	C24—C22—C32	107.7 (2)
O2—C4—C5	105.3 (3)	C23—C22—C32	115.1 (2)
C3—C4—C5	117.6 (4)	C24—C22—C16	108.1 (2)
O2—C4—H4	108.5	C23—C22—C16	112.2 (2)
C3—C4—H4	108.5	C32—C22—C16	106.5 (2)
C5—C4—H4	108.5	C22—C23—H23A	109.5
C4—C5—C6	105.0 (3)	C22—C23—H23B	109.5
C4—C5—H5A	110.7	H23A—C23—H23B	109.5
C6—C5—H5A	110.7	C22—C23—H23C	109.5
C4—C5—H5B	110.7	H23A—C23—H23C	109.5
C6—C5—H5B	110.7	H23B—C23—H23C	109.5
H5A—C5—H5B	108.8	C25—C24—C22	113.0 (3)
C7—C6—C5	103.0 (3)	C25—C24—H24A	109.0
C7—C6—H6A	111.2	C22—C24—H24A	109.0
C5—C6—H6A	111.2	C25—C24—H24B	109.0
C7—C6—H6B	111.2	C22—C24—H24B	109.0
C5—C6—H6B	111.2	H24A—C24—H24B	107.8
H6A—C6—H6B	109.1	C26—C25—C24	110.2 (3)
O2—C7—C6	101.8 (2)	C26—C25—H25A	109.6
O2—C7—C36	108.9 (3)	C24—C25—H25A	109.6
C6—C7—C36	112.3 (3)	C26—C25—H25B	109.6
O2—C7—C8	104.7 (3)	C24—C25—H25B	109.6
C6—C7—C8	115.2 (3)	H25A—C25—H25B	108.1
C36—C7—C8	112.8 (3)	O7—C26—C25	107.9 (3)
C7—C8—C19	115.9 (3)	O7—C26—C29	107.2 (2)
C7—C8—C9	110.7 (3)	C25—C26—C29	116.4 (2)
C19—C8—C9	104.2 (2)	O7—C26—H26	108.4
C7—C8—H8	108.6	C25—C26—H26	108.4
C19—C8—H8	108.6	C29—C26—H26	108.4
C9—C8—H8	108.6	O8—C27—O7	124.0 (3)
C10—C9—C8	105.9 (3)	O8—C27—C28	125.0 (3)
C10—C9—H9A	110.5	O7—C27—C28	111.0 (4)
C8—C9—H9A	110.5	C27—C28—H28A	109.5
C10—C9—H9B	110.5	C27—C28—H28B	109.5
C8—C9—H9B	110.5	H28A—C28—H28B	109.5
H9A—C9—H9B	108.7	C27—C28—H28C	109.5
C9—C10—C11	103.5 (3)	H28A—C28—H28C	109.5
C9—C10—H10A	111.1	H28B—C28—H28C	109.5
C11—C10—H10A	111.1	C30—C29—C26	111.6 (2)
C9—C10—H10B	111.1	C30—C29—C31	109.0 (2)
C11—C10—H10B	111.1	C26—C29—C31	104.9 (2)
H10A—C10—H10B	109.0	C30—C29—C32	112.8 (2)
C10—C11—C12	106.2 (2)	C26—C29—C32	107.3 (2)
C10—C11—C19	100.8 (2)	C31—C29—C32	111.0 (2)
C12—C11—C19	110.8 (2)	C29—C30—H30A	109.5
C10—C11—C13	116.6 (2)	C29—C30—H30B	109.5
C12—C11—C13	112.5 (2)	H30A—C30—H30B	109.5
C19—C11—C13	109.2 (2)	C29—C30—H30C	109.5
C11—C12—H12A	109.5	H30A—C30—H30C	109.5
C11—C12—H12B	109.5	H30B—C30—H30C	109.5
H12A—C12—H12B	109.5	C29—C31—H31A	109.5
C11—C12—H12C	109.5	C29—C31—H31B	109.5
H12A—C12—H12C	109.5	H31A—C31—H31B	109.5
H12B—C12—H12C	109.5	C29—C31—H31C	109.5
C15—C13—C14	107.6 (2)	H31A—C31—H31C	109.5
C15—C13—C16	108.3 (2)	H31B—C31—H31C	109.5
C14—C13—C16	112.7 (2)	C33—C32—C29	115.3 (2)
C15—C13—C11	111.1 (2)	C33—C32—C22	108.2 (2)
C14—C13—C11	110.2 (2)	C29—C32—C22	116.8 (2)
C16—C13—C11	106.9 (2)	C33—C32—H32	105.1
C13—C14—H14A	109.5	C29—C32—H32	105.1
C13—C14—H14B	109.5	C22—C32—H32	105.1
H14A—C14—H14B	109.5	O5—C33—C15	107.8 (2)
C13—C14—H14C	109.5	O5—C33—C32	111.6 (2)
H14A—C14—H14C	109.5	C15—C33—C32	110.8 (2)
H14B—C14—H14C	109.5	O5—C33—H33	108.9
C33—C15—C13	113.5 (2)	C15—C33—H33	108.9
C33—C15—H15A	108.9	C32—C33—H33	108.9
C13—C15—H15A	108.9	O6—C34—O5	122.8 (3)
C33—C15—H15B	108.9	O6—C34—C35	125.1 (4)
C13—C15—H15B	108.9	O5—C34—C35	112.1 (3)
H15A—C15—H15B	107.7	C34—C35—H35A	109.5
C17—C16—C13	111.1 (2)	C34—C35—H35B	109.5
C17—C16—C22	115.1 (2)	H35A—C35—H35B	109.5
C13—C16—C22	115.8 (2)	C34—C35—H35C	109.5
C17—C16—H16	104.4	H35A—C35—H35C	109.5
C13—C16—H16	104.4	H35B—C35—H35C	109.5
C22—C16—H16	104.4	C7—C36—H36A	109.5
C18—C17—C16	113.1 (2)	C7—C36—H36B	109.5
C18—C17—H17A	109.0	H36A—C36—H36B	109.5
C16—C17—H17A	109.0	C7—C36—H36C	109.5
C18—C17—H17B	109.0	H36A—C36—H36C	109.5
C16—C17—H17B	109.0	H36B—C36—H36C	109.5
H17A—C17—H17B	107.8	C3—O1—H1	109.5
O3—C18—C19	108.9 (2)	C7—O2—C4	109.6 (2)
O3—C18—C17	108.6 (2)	C20—O3—C18	118.5 (2)
C19—C18—C17	110.6 (2)	C34—O5—C33	118.0 (2)
O3—C18—H18	109.6	C27—O7—C26	119.3 (3)
			
O1—C3—C4—O2	−65.8 (4)	C9—C8—C19—C18	142.2 (3)
C1—C3—C4—O2	56.1 (4)	C7—C8—C19—C11	139.9 (3)
C2—C3—C4—O2	177.9 (3)	C9—C8—C19—C11	18.0 (3)
O1—C3—C4—C5	53.1 (5)	C17—C16—C22—C24	−56.5 (3)
C1—C3—C4—C5	175.1 (4)	C13—C16—C22—C24	171.6 (2)
C2—C3—C4—C5	−63.1 (5)	C17—C16—C22—C23	61.3 (3)
O2—C4—C5—C6	4.7 (4)	C13—C16—C22—C23	−70.6 (3)
C3—C4—C5—C6	−115.7 (4)	C17—C16—C22—C32	−172.0 (2)
C4—C5—C6—C7	−26.2 (4)	C13—C16—C22—C32	56.2 (3)
C5—C6—C7—O2	37.8 (4)	C23—C22—C24—C25	69.1 (3)
C5—C6—C7—C36	−78.5 (4)	C32—C22—C24—C25	−55.2 (3)
C5—C6—C7—C8	150.5 (3)	C16—C22—C24—C25	−169.9 (3)
O2—C7—C8—C19	−177.4 (3)	C22—C24—C25—C26	58.4 (4)
C6—C7—C8—C19	71.7 (4)	C24—C25—C26—O7	−177.0 (3)
C36—C7—C8—C19	−59.1 (4)	C24—C25—C26—C29	−56.5 (4)
O2—C7—C8—C9	−59.1 (3)	O7—C26—C29—C30	47.0 (3)
C6—C7—C8—C9	−170.0 (3)	C25—C26—C29—C30	−73.8 (3)
C36—C7—C8—C9	59.3 (4)	O7—C26—C29—C31	−70.9 (3)
C7—C8—C9—C10	−115.6 (3)	C25—C26—C29—C31	168.3 (3)
C19—C8—C9—C10	9.7 (3)	O7—C26—C29—C32	170.9 (2)
C8—C9—C10—C11	−34.2 (3)	C25—C26—C29—C32	50.1 (3)
C9—C10—C11—C12	−71.1 (3)	C30—C29—C32—C33	−54.0 (3)
C9—C10—C11—C19	44.5 (3)	C26—C29—C32—C33	−177.2 (2)
C9—C10—C11—C13	162.5 (2)	C31—C29—C32—C33	68.7 (3)
C10—C11—C13—C15	66.1 (3)	C30—C29—C32—C22	74.7 (3)
C12—C11—C13—C15	−57.1 (3)	C26—C29—C32—C22	−48.5 (3)
C19—C11—C13—C15	179.4 (2)	C31—C29—C32—C22	−162.5 (2)
C10—C11—C13—C14	−53.1 (3)	C24—C22—C32—C33	−175.9 (2)
C12—C11—C13—C14	−176.3 (2)	C23—C22—C32—C33	64.8 (3)
C19—C11—C13—C14	60.2 (3)	C16—C22—C32—C33	−60.2 (3)
C10—C11—C13—C16	−175.9 (2)	C24—C22—C32—C29	52.0 (3)
C12—C11—C13—C16	61.0 (3)	C23—C22—C32—C29	−67.3 (3)
C19—C11—C13—C16	−62.5 (3)	C16—C22—C32—C29	167.7 (2)
C14—C13—C15—C33	−71.5 (3)	C13—C15—C33—O5	177.4 (2)
C16—C13—C15—C33	50.6 (3)	C13—C15—C33—C32	−60.3 (3)
C11—C13—C15—C33	167.8 (2)	C29—C32—C33—O5	−42.7 (3)
C15—C13—C16—C17	175.7 (2)	C22—C32—C33—O5	−175.6 (2)
C14—C13—C16—C17	−65.3 (3)	C29—C32—C33—C15	−162.8 (2)
C11—C13—C16—C17	55.9 (3)	C22—C32—C33—C15	64.3 (3)
C15—C13—C16—C22	−50.6 (3)	C6—C7—O2—C4	−37.0 (3)
C14—C13—C16—C22	68.4 (3)	C36—C7—O2—C4	81.8 (3)
C11—C13—C16—C22	−170.4 (2)	C8—C7—O2—C4	−157.3 (3)
C13—C16—C17—C18	−53.3 (3)	C3—C4—O2—C7	147.0 (3)
C22—C16—C17—C18	172.6 (2)	C5—C4—O2—C7	20.5 (4)
C16—C17—C18—O3	174.6 (2)	O4—C20—O3—C18	−6.4 (5)
C16—C17—C18—C19	55.1 (3)	C21—C20—O3—C18	174.9 (3)
O3—C18—C19—C11	−179.3 (2)	C19—C18—O3—C20	−153.5 (3)
C17—C18—C19—C11	−60.1 (3)	C17—C18—O3—C20	86.0 (3)
O3—C18—C19—C8	57.8 (3)	O6—C34—O5—C33	6.2 (5)
C17—C18—C19—C8	177.0 (3)	C35—C34—O5—C33	−173.2 (3)
C10—C11—C19—C18	−171.2 (2)	C15—C33—O5—C34	−97.3 (3)
C12—C11—C19—C18	−59.0 (3)	C32—C33—O5—C34	140.9 (3)
C13—C11—C19—C18	65.5 (3)	O8—C27—O7—C26	0.4 (6)
C10—C11—C19—C8	−38.6 (3)	C28—C27—O7—C26	−179.6 (3)
C12—C11—C19—C8	73.6 (3)	C25—C26—O7—C27	−100.7 (4)
C13—C11—C19—C8	−161.9 (2)	C29—C26—O7—C27	133.3 (3)
C7—C8—C19—C18	−96.0 (3)		

### Hydrogen-bond geometry (Å, °)

**Table:**

*D*—H···*A*	*D*—H	H···*A*	*D*···*A*	*D*—H···*A*
O1—H1···O8^i^	0.82	2.36	3.050 (4)	142
C8—H8···O8^i^	0.98	2.58	3.526 (4)	161
C35—H35C···O6^ii^	0.96	2.45	3.403 (5)	172

Symmetry codes: (i) *x*+1/2, −*y*+3/2, −*z*+2; (ii) *x*+1/2, −*y*+1/2, −*z*+2.
